# Prevalence and Determinants of Osteoporosis Among Patients Undergoing Dual-Energy X-ray Absorptiometry (DXA) Scanning at a Tertiary Hospital in Damascus, Syria: A Cross-Sectional Study

**DOI:** 10.7759/cureus.113489

**Published:** 2026-07-28

**Authors:** Fatema Nserat, Chahed Manssour, Taghrid Hammoud

**Affiliations:** 1 Medicine, Syrian Private University, Damascus, SYR; 2 Internal Medicine, Faculty of Medicine, Syrian Private University, Damascus, SYR

**Keywords:** bone mineral density, dual-energy x-ray absorptiometry, osteoporosis, prevalence, risk factors

## Abstract

Background: This study aimed to determine the prevalence of osteoporosis and identify factors associated with osteoporosis among adult patients undergoing dual-energy X-ray absorptiometry (DXA) at the National Hospital in Damascus, Syria.

Methods: A cross-sectional study was conducted at the National Hospital in Damascus, Syria, enrolling 158 adult patients referred for DXA evaluation. Bone mineral density (BMD) was classified according to World Health Organization (WHO) criteria based on the lowest T-score from lumbar spine or femoral neck measurements. Data on demographic characteristics, reproductive history, lifestyle factors, comorbidities, and medication use were collected from structured clinical evaluation forms. Statistical analyses included chi-square tests (with Fisher’s exact or Monte Carlo correction when appropriate) and Spearman’s correlation.

Results: The mean age was 60.79 ± 6.56 years, and 86.7% of the participants were female. Osteoporosis was the most prevalent diagnosis, affecting 53.2% (n = 84) of participants, followed by osteopenia in 36.7% (n = 58), while 10.1% (n = 16) had normal BMD. Female sex was significantly associated with osteoporosis (χ²(2) = 11.65, p = 0.003). Hyperthyroidism was also significantly associated with osteoporosis (p = 0.025). Body mass index (BMI) showed a significant negative correlation with femoral T-score (r = -0.257, p = 0.001). No significant associations were observed with age, smoking, diabetes mellitus, hypertension, or steroid use; however, marital status, cervical disc disease, and lumbar disc disease showed significant associations with osteoporosis classification.

Conclusions: Osteoporosis was identified in more than half of the patients undergoing DXA evaluation in this study, while osteopenia affected 36.7% of participants, highlighting the need for early identification and preventive strategies. Female sex and hyperthyroidism were significantly associated with osteoporosis. These findings may help characterize osteoporosis among patients referred for DXA evaluation at this center and may support future screening and preventive strategies.

## Introduction

Osteoporosis represents a significant global health challenge, characterized by diminished bone mineral density (BMD) and increased susceptibility to fragility fractures, which substantially impact patient quality of life, healthcare systems, and societal resources [1]. The World Health Organization (WHO) established diagnostic criteria in 1994 defining osteoporosis as a BMD T-score of -2.5 or below and osteopenia (low bone mass) as a T-score between -1.0 and -2.5, using dual-energy X-ray absorptiometry (DXA) as the reference standard [2]. These criteria, originally developed for postmenopausal women, have since been extended to men aged 50 years and above, as well as perimenopausal women, facilitating epidemiological comparisons across populations [3].

BMD measurement by DXA remains the gold standard for noninvasive assessment of skeletal health, serving as the single best predictor of fracture risk in older adults without prior fracture [4]. The prevalence of osteoporosis varies considerably across populations, influenced by demographic characteristics, genetic factors, environmental conditions, comorbidities, and healthcare access [5]. Globally, systematic reviews have reported substantial regional variations, with prevalence rates of 38.5% in Africa, 18.6% in Europe, 16.7% in Asia, 12.4% in the Americas, and 13.5% in Australia [6].

The burden of osteoporosis in the Middle East and North Africa region has gained increasing recognition [7]. In Saudi Arabia, recent studies have reported osteoporosis prevalence ranging from 23.4% to 39.5% among adults, with significant regional variations [8,9]. A large-scale study in Al-Ahsa found an overall prevalence of 31.54%, higher among women than men [7]. Similarly, research from Riyadh demonstrated femoral and lumbar spine osteoporosis prevalence predominantly affecting older women [9]. In Pakistan, osteoporosis affects millions with increasing projections [10]. Studies utilizing DXA have revealed overall osteoporosis prevalence of 38.5%, with site-specific rates of 38.7% in the lumbar spine, 8.9% in the hip, and 38.4% in the forearm [11].

Despite the substantial burden of osteoporosis worldwide, the prevalence and determinants of this condition in conflict-affected settings remain poorly characterized. The National Hospital in Damascus serves as a major tertiary healthcare facility for the Damascus governorate and surrounding regions, providing specialized care in endocrinology, rheumatology, orthopedics, neurology, and internal medicine. Understanding the epidemiology of osteoporosis in this setting is essential for developing targeted screening strategies and treatment protocols appropriate for the local context.

Several factors contribute to osteoporosis risk, including age, sex, menopausal status, body mass index (BMI), lifestyle factors, comorbidities, and medication use [5,8,12]. Comorbidities, including chronic kidney disease and endocrine disorders, may also influence bone health [13]. Women are at particularly elevated risk due to the accelerated bone loss associated with menopause, with studies consistently demonstrating higher osteoporosis prevalence among females compared to males [8,14]. In Saudi Arabia, women exhibited a prevalence of 36.7% compared to 21.2% in men [8], while international data suggest global rates of 35.3% in women versus 12.5% in men [6]. BMI has been inversely associated with osteoporosis risk in multiple studies, with lower BMI correlating with reduced BMD [8]. Comorbidities, including chronic kidney disease and endocrine disorders, may also influence bone health [13]. Furthermore, medication use, particularly corticosteroids, represents an established risk factor for secondary osteoporosis [3,15].

The present study addresses a critical knowledge gap regarding osteoporosis epidemiology in Syria. To our knowledge, no previous large-scale investigation has systematically evaluated the prevalence and determinants of osteoporosis using DXA in a Syrian population. Given the high prevalence of osteoporosis in neighboring Middle Eastern countries and the unique challenges facing the Syrian healthcare system, and given the limited data regarding osteoporosis prevalence and associated factors in Syria, this study was primarily exploratory and aimed to characterize osteoporosis prevalence and identify demographic and clinical factors associated with BMD classification. Specifically, we aimed to (1) determine the prevalence of osteoporosis and osteopenia among patients referred for DXA scanning; (2) identify demographic, reproductive, lifestyle, and clinical factors associated with osteoporosis classification; and (3) examine correlations between clinical variables and BMD measurements at the lumbar spine and femoral neck.

## Materials and methods

Study design and setting

This was a cross-sectional study with prospective data collection conducted at the National Hospital in Damascus, Syria. Adult patients referred for DXA examination were consecutively recruited during the study period from April 2026 to June 2026. Data were collected through face-to-face interviews using a structured questionnaire, and DXA results obtained during the same visit were recorded for analysis.

 Data were collected prospectively from April 2026 to June 2026.

Study population

The study included consecutive adult patients (≥18 years) referred for DXA examination during the study period. All eligible patients who underwent DXA scanning during the data collection period were invited to participate. Participants were enrolled after providing informed consent. A formal sample size calculation was not performed because all eligible patients undergoing DXA scanning during the study period were consecutively included.

Inclusion Criteria

Patients were included if they met the following criteria: (1) age 18 years or older at the time of DXA scanning, (2) availability of complete DXA T-scores for both lumbar spine (L1-L4) and femoral neck, and (3) complete demographic data including age and sex.

Exclusion Criteria

Patients were excluded if (1) age was below 18 years, (2) DXA scan data were incomplete (missing T-scores for either site), or (3) essential demographic information was unavailable.

Variables and measurements

Outcome Variable

BMD was measured using a Hologic Discovery Wi DXA scanner (Hologic Inc., Marlborough, MA, USA). Daily quality control was performed using a calibration phantom according to the manufacturer’s recommendations to ensure measurement accuracy.

The primary outcome was the BMD status classified according to the WHO criteria based on the lowest T-score obtained from either the lumbar spine or femoral neck measurement [1]. The classification was defined as follows: normal (T-score ≥ -1.0), osteopenia (T-score between -2.5 and -1.0), and osteoporosis (T-score ≤ -2.5). 

Independent Variables

The independent variables included demographic characteristics, reproductive history, lifestyle factors, comorbidities, and medication use. Demographic, reproductive, and lifestyle information was collected prospectively from participants through a structured questionnaire administered by the investigator during the DXA visit. Clinical diagnoses, medication history, and DXA findings were verified using the participants’ medical records and DXA reports.

Demographic variables: Age (continuous, in years), sex (male/female), marital status (married/single/divorced), BMI calculated as weight in kilograms divided by height in meters squared (continuous, kg/m²), and place of residence (Damascus, Rif-Damascus, or other). Occupation was categorized as unemployed, bureaucratic, or manual work and was treated as an ordinal variable according to expected physical activity level, with manual work considered higher physical demand compared with bureaucratic work and unemployment.

Reproductive history (females only): Menopausal status (premenopausal vs. postmenopausal), age at menopause (continuous, years), number of deliveries (continuous), and number of children (continuous).

Lifestyle factors: Smoking status (current smoker: yes/no), with additional information on smoking quantity and duration collected when applicable.

Comorbidities: Hypertension, diabetes mellitus, heart disease, hypothyroidism, hyperthyroidism, hyperparathyroidism, asthma, chronic kidney disease, rheumatoid arthritis, cancer, previous fractures, cervical disc disease, lumbar disc disease, and previously diagnosed osteoporosis were recorded as binary variables (present/absent) based on participant interviews and documented clinical diagnoses.

Medication history: Current use of corticosteroids (prednisone, hydrocortisone, or other systemic corticosteroids), vitamin D supplements, calcium supplements, and calcium channel blockers was recorded as binary variables (yes/no). Medication information was collected through participant interviews and verified using available medical records when possible.

Data collection and management

Data were collected prospectively using a structured questionnaire administered by the investigator through face-to-face interviews with participants during their DXA visit. The questionnaire was developed based on previously reported osteoporosis risk factors and included standardized assessment of demographic, reproductive, lifestyle, and clinical variables, including marital status, smoking history, reproductive history, physical activity-related occupation, and medication use. Additional clinical information and DXA findings were recorded from the routine clinical assessment performed on the same day.

Statistical analysis

All statistical analyses were performed using IBM SPSS Statistics for Windows, version 25.0 (released 2017, IBM Corp., Armonk, NY). Descriptive statistics were computed for all variables: means and standard deviations for continuous variables, and frequencies with percentages for categorical variables. The normality of continuous variables was assessed using the Shapiro-Wilk test.

For bivariate analyses, associations between categorical variables and osteoporosis classification (normal, osteopenia, osteoporosis) were examined using chi-square tests of independence. Fisher’s exact test or Monte Carlo simulation was applied when expected cell counts were insufficient to meet the assumptions of the chi-square test, depending on the contingency table structure. The strength of associations was assessed using Cramer's V for categorical variables.

Cramer’s V values of 0.10, 0.30, and 0.50 were interpreted as indicating small, medium, and large effect sizes, respectively. For continuous independent variables, Spearman's rank correlation coefficient (ρ) was used to examine relationships with osteoporosis classification, as the outcome variable was ordinal (normal, osteopenia, and osteoporosis). Occupation categories were analyzed as an ordinal variable based on expected physical activity level, with manual work considered higher physical demand than bureaucratic work and unemployment. Spearman's correlation was also employed to investigate associations between continuous clinical variables (age, BMI, postmenopausal duration, pack-years, number of pregnancies, and number of children) and both spinal and femoral T-scores.

Missing data were handled using complete-case analysis, and participants with missing values for specific variables were excluded from the corresponding analyses.

This study was reported in accordance with the STROBE (Strengthening the Reporting of Observational Studies in Epidemiology) statement for cross-sectional studies [14]. All relevant items from the STROBE checklist were followed to ensure transparent and complete reporting of the study methodology and findings.

Ethical considerations

Ethical approval was granted by the Institutional Review Board of Syrian Private University, Syria (IRB approval no. MD-30032026-153). Participation was voluntary; electronic informed consent was obtained from all participants prior to data collection. All responses were anonymous. The study adhered to the Declaration of Helsinki.

## Results

Participant characteristics

A total of 158 participants were included in the study. The mean age was 60.79 ± 6.56 years, with a mean postmenopausal duration of 14.52 ± 7.29 years. The mean BMI was 29.47 ± 5.55 kg/m². The average smoking exposure was 7.29 ± 8.59 pack-years. Most participants were female (86.7%, n = 137), married (91.1%, n = 144), unemployed (67.7%, n = 107), and residing in Damascus (60.8%, n = 96). Postmenopausal women constituted (81.0%, n = 111) of the study population.

Regarding comorbidities, hypertension was the most common condition (36.1%, n = 57), diabetes mellitus (19.0%, n = 30), hyperthyroidism (16.5%, n = 26), hypothyroidism (14.6%, n = 23), previous fractures (14.6%, n = 23), and steroid use (13.3%, n = 21). Other conditions, including chronic kidney disease, hyperparathyroidism, rheumatoid arthritis, and cancer, were less frequently reported.

**Figure 1:**
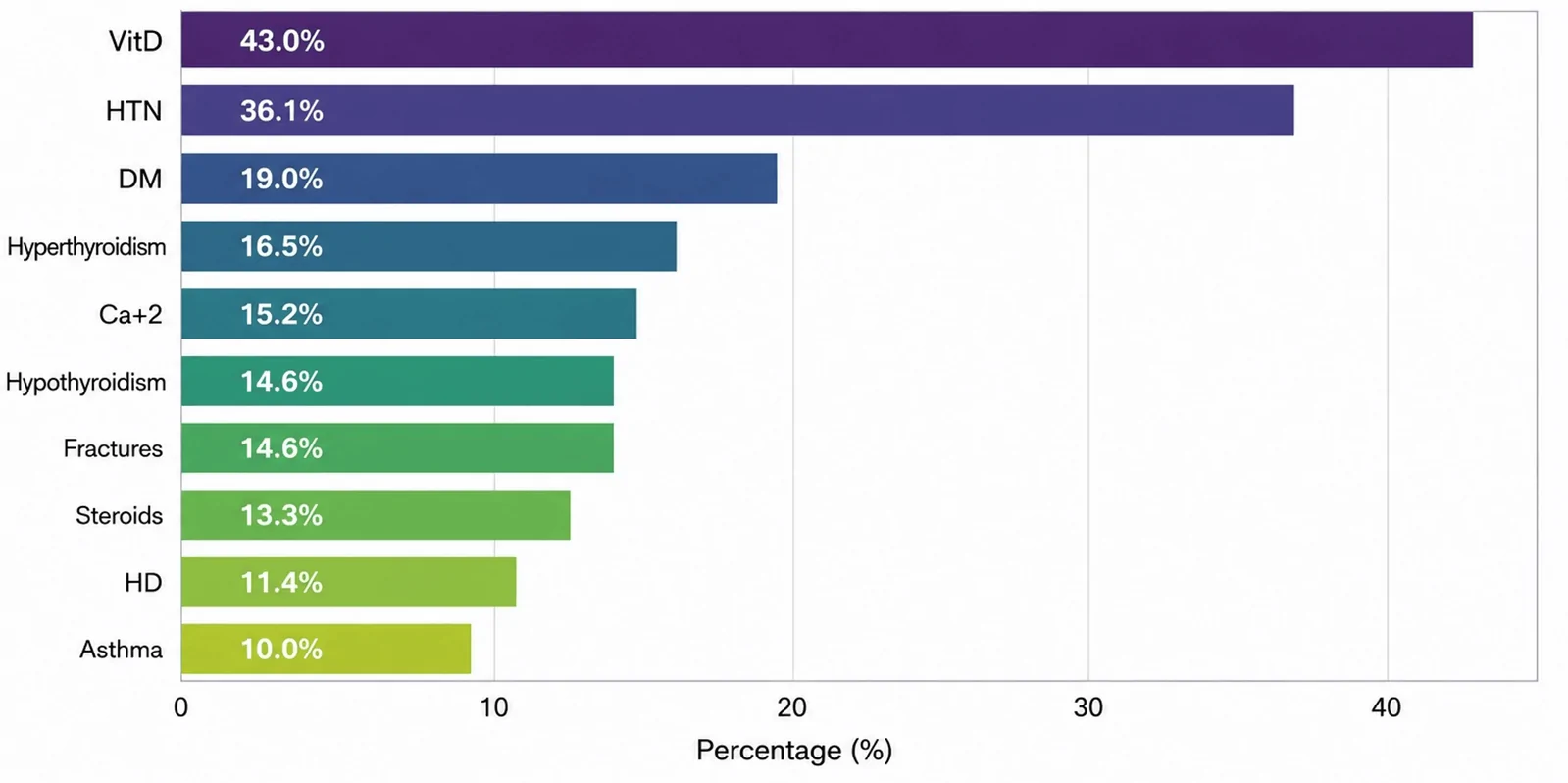
Prevalence of comorbidities and supplement use Vit D: vitamin D supplements; HTN: hypertension; DM: diabetes mellitus; HD: heart disease; Ca+2: calcium supplements

Regarding medication history, vitamin D supplementation was the most commonly reported medication (43.0%, n = 68), followed by calcium supplementation (15.2%, n = 24) and steroid use (13.3%, n = 21) (Figure 1).

Based on the BMD assessment, osteoporosis was the most prevalent diagnosis, affecting 53.2% (n = 84) of the participants, followed by osteopenia in 36.7% (n = 58), while only 10.1% (n = 16) had normal bone density. The mean spinal and femoral T-scores were -3.10 ± 1.05 and -1.91 ± 0.97, respectively. The mean spinal and femoral Z-scores were -1.72 ± 1.12 and -1.18 ± 0.89, respectively (Table 1).

**Table 1:** Main results Menopausal status (female participants, n = 137)

Characteristic	n	%
Sex		
Female	137	86.7
Male	21	13.3
Marital status		
Married	144	91.1
Single	13	8.2
Divorced	1	0.6
Occupation		
Unemployed	107	67.7
Bureaucratic job	48	30.4
Muscular (manual) job	3	1.9
Region		
Damascus	96	60.8
Rural Damascus	45	28.5
Daraa	5	3.2
Al-Hasakah	5	3.2
Deir ez-Zor	2	1.3
Quneitra	2	1.3
Homs	1	0.6
Tartus	1	0.6
Other	1	0.6
Menopausal statusᵃ		
Postmenopausal	111	81
Premenopausal	26	19
Comorbidities and risk factors		
Smoking	49	31.0
Hypertension (HTN)	57	36.1
Diabetes mellitus (DM)	30	19.0
Heart disease (HD)	18	11.4
Hypothyroidism	23	14.6
Hyperthyroidism	26	16.5
Hyperparathyroidism	12	7.6
Asthma	16	10.1
Osteoporosis (known history)	6	3.8
Rheumatoid arthritis	3	1.9
Cancer	2	1.3
Chronic kidney disease (CKD)	4	2.5
Previous fractures	23	14.6
Cervical disc disease	9	5.7
Lumbar disc disease	19	12.0
Medications and supplements		
Vitamin D (VitD)	68	43.0
Calcium (Ca²⁺)	24	15.2
Steroids	21	13.3
Calcium channel blockers (CCB)	5	3.2
Osteoporosis classification		
Normal	16	10.1
Osteopenia	58	36.7
Osteoporosis	84	53.2
Continuous variables	Mean	SD
Age (years)	60.79	6.56
Years since menopause	14.52	7.29
Pack-years (smokers)	7.29	8.59
Spinal T-scoreᵇ	−3.10	1.05
Femoral T-scoreᵇ	−1.91	0.97
Spinal Z-scoreᵇ	−1.72	1.12
Femoral Z-scoreᵇ	−1.18	0.89
Body mass index (kg/m²)	29.47	5.55

Factors associated with BMD classification

**Figure 2:**
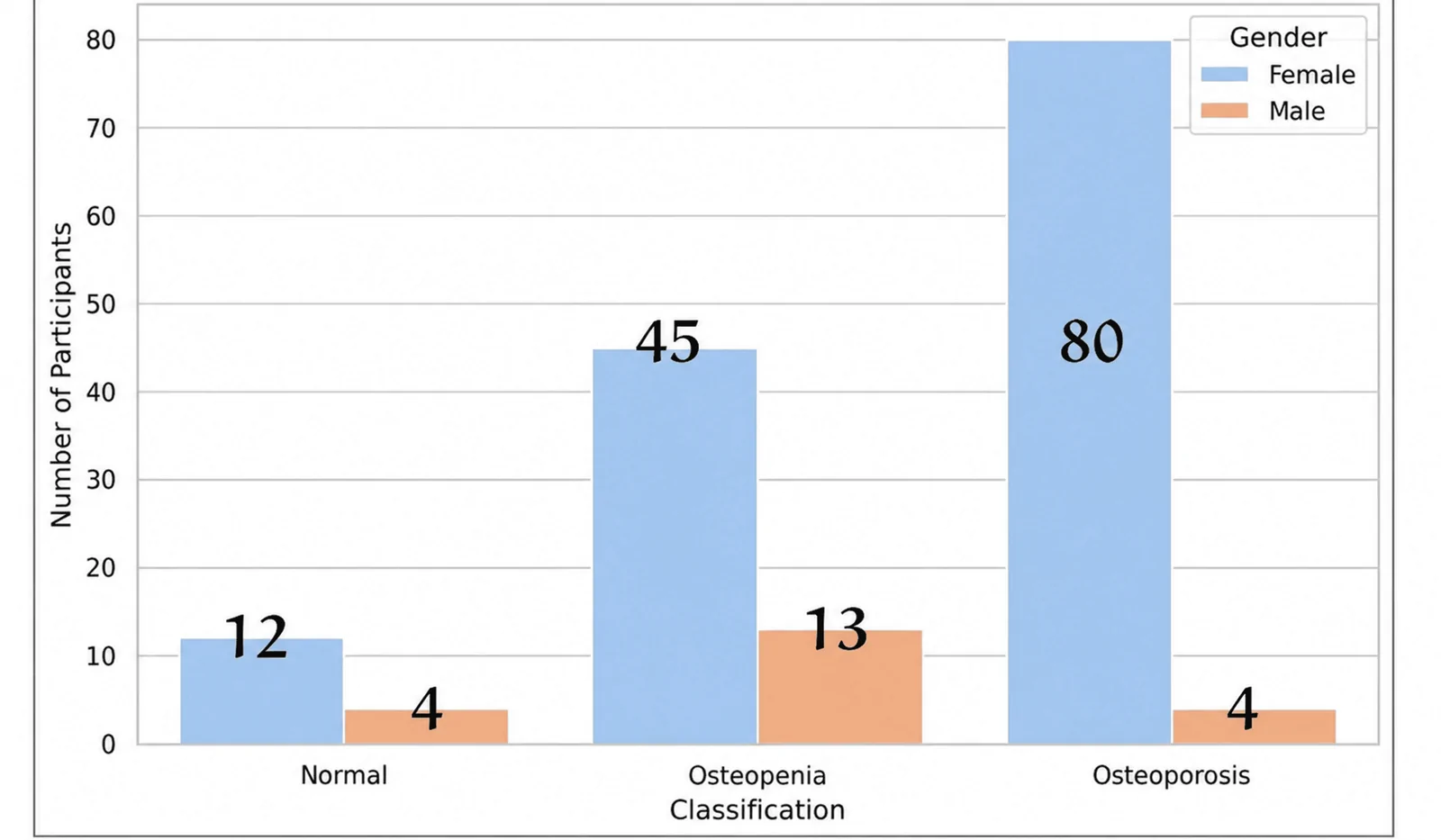
BMD classification distribution by gender Comparison of bone mineral density (BMD) classifications between the male and female participants (p = 0.003).

The distribution of osteoporosis classifications differed significantly according to sex (χ²(2) = 11.65, p = 0.003). Females represented 95.2% of the osteoporosis group compared with 77.6% of the osteopenia group and 75.0% of the normal group, indicating a higher burden of osteoporosis among women (Figure 2). 

Marital status was also significantly associated with osteoporosis classification (Fisher’s exact test, p = 0.012; Cramer’s V = 0.203). Married individuals constituted the majority of participants across all categories. Although single individuals represented 11.9% of the osteoporosis group compared with 1.7% of the osteopenia group, the highest proportion of single individuals was observed in the normal BMD group (12.5%).

No significant association was observed between osteoporosis classification and the muscular effort required in occupation (Spearman’s ρ = -0.035, p = 0.666), suggesting a negligible effect size. Similarly, geographical region was not associated with bone density status (Monte Carlo corrected chi-square = 10.74, df = 8, p = 0.701).

Although postmenopausal women were more frequently represented in the osteopenia (82.2%) and osteoporosis (83.8%) groups than in the normal group (58.3%), this difference did not reach statistical significance (p = 0.108). Among the assessed comorbidities, hyperthyroidism demonstrated a significant association with osteoporosis classification (χ²(2) = 7.38, p = 0.025; Cramer's V = 0.216). The highest prevalence of hyperthyroidism was observed in the osteoporosis group (23.8%), compared with 12.5% in the normal group and 6.9% in the osteopenia group.

Hypothyroidism showed a borderline, non-significant association with osteoporosis status (χ²(2) = 5.86, p = 0.053; Cramer's V = 0.193), with prevalence increasing from 0% in the normal group to 20.5% in the osteoporosis group. Cervical disc disease was significantly associated with bone density classification (χ²(2) = 6.04, p = 0.029; Cramer's V = 0.196), being most prevalent among participants with osteopenia (12.1%) and less common among those with osteoporosis (2.4%). Likewise, lumbar disc disease demonstrated a significant association (χ²(2) = 9.44, p = 0.009; Cramer's V = 0.244), with the highest prevalence observed in the osteopenia group (22.4%) compared with the normal (6.3%) and osteoporosis (6.0%) groups.

No statistically significant associations were found between osteoporosis classification and hypertension (p = 0.958), smoking status (p = 0.513), diabetes mellitus (p = 0.452), heart disease (p = 0.128), asthma (p = 0.281), previously diagnosed osteoporosis (p = 0.649), Rheumatoid arthritis (p = 0.809), cancer (p = 0.858), vitamin D supplementation (p = 0.494), calcium supplementation (p = 0.476), steroid use (p = 0.679), history of fractures (p = 0.175), calcium channel blocker use (p = 0.446), hyperparathyroidism (p = 0.084), or chronic kidney disease (p = 0.733).

Continuous variables were also examined. BMI was not significantly associated with osteoporosis classification (Spearman's ρ = -0.097, p = 0.225), indicating a weak negative relationship. Similarly, age (ρ = 0.051, p = 0.522), postmenopausal duration (ρ = 0.062, p = 0.516), smoking exposure measured by pack-years (ρ = 0.015, p = 0.923), number of pregnancies (ρ = 0.063, p = 0.474), and number of children (ρ = 0.063, p = 0.474) were not significantly related to osteoporosis classification, with all effect sizes being negligible (all |ρ| < 0.10) (Table 2).

**Table 2:** Affecting factors HTN: hypertension; DM: diabetes mellitus; HD: heart disease; Op: osteoporosis; Vit D: vitamin D supplementation; Ca+2: calcium supplementation; CCB: calcium channel blocker; CKD: chronic kidney disease; BMI: body mass index * Percentages for menopausal status were calculated using female participants within each osteoporosis category as the denominator.

		Normal	Osteopenia	Osteoporosis	Test	P-value
Sex						
	Female	12 (75%)	45 (77.6%)	80 (95.2%)	Chi-square	0.003
	Male	4 (25%)	13 (22.4%)	4 (4.8%)		
Marital status						
	Married	13 (81.3%)	57 (98.3%)	74 (88.1%)	Exact test	0.012
	Single	2 (12.5%)	1 (1.7%)	10 (11.9)		
	Divorced	1 (6.3%)	0	0		
Occupation						
	Unemployed	11 (68.8%)	38 (65.5%)	58 (69%)	Spearman (R=-0.035)	0.666
	Bureaucratic job	5 (31.3%)	17 (29.3%)	26 (31%)		
	Muscular job	0	3 (5.2%)	0		
Region						
	Damascus	13 (81.3%)	35 (60.3%)	48 (57.1%)	Chi-square with Montecarlo transformation (with 10,000 simulations and 99% confidence)	0.701
	Rural Damascus	2 (12.5%)	19 (32.8%)	24 (28.6%)		
	Daraa	0	1 (1.7%)	4 (4.8%)		
	Al-Hasakah	1 (6.3%)	2 (3.4%)	2 (2.4%)		
	Deir ez-Zor	0	0	2 (2.4%)		
	Homs	0	0	1 (1.2%)		
	Quneitra	0	0	2 (2.4%)		
	Tartus	0	1 (1.7%)	0		
	Other	0	0	1 (1.2%)		
Menopausal status						
	Postmenopausal	7 (58.3%)	37 (82.2%)	67 (83.8%)	Chi-square	0.108
	Premenopausal	5 (41.7%)	8 (17.8%)	13 (16.3%)		
HTN		6 (40%)	21 (36.2%)	30 (36.1%)	Chi-square	0.958
Smoking		3 (18.8%)	18 (31%)	28 (33.3%)	Chi-square	0.513
DM		3 (18.8%)	14 (24.1%)	13 (15.7%)	Chi-square	0.452
HD		4 (25%)	4 (6.9%)	10 (12%)	Chi-square	0.128
Hypothyroidism		0	6 (10.3%)	17 (20.5%)	Chi-square	0.053
Hyperthyroidism		2 (12.5%)	4 (6.9%)	20 (23.8%)	Chi-square	0.025
Asthma		2 (12.5%)	3 (5.2%)	11 (13.3%)	Chi-square	0.281
OP		0	2 (3.4%)	4 (4.8%)	Chi-square	0.649
Rheumatoid arthritis		0	1 (1.7%)	2 (2.4%)	Fisher’s exact test (Monte Carlo correction)	0.809
Cancer		0	1 (1.7%)	1 (1.2%)	Fisher’s exact test (Monte Carlo correction)	0.858
VitD		9 (56.3%)	23 (39.7%)	36 (42.9%)	Chi-square	0.494
Ca+2		4 (25%)	9 (15.5%)	11 (13.1%)	Chi-square	0.476
Steroids		1 (6.3%)	8 (13.8%)	12 (14.3%)	Chi-square	0.679
Fractures		0	8 (13.8%)	15 (17.9%)	Chi-square	0.175
Cervical Disk		0	7 (12.1%)	2 (2.4%)	Fisher’s exact test (Monte Carlo correction)	0.029
Lumbar Disk		1 (6.3%)	13 (22.4%)	5 (6%)	Fisher’s exact test (Monte Carlo correction)	0.009
CCB		0	1 (1.7%)	4 (4.8%)	Chi-square	0.446
Hyperparathyroidism		0	2 (3.4%)	10 (11.9%)	Chi-square	0.084
CKD		0	2 (3.4%)	2 (2.4%)	Fisher’s exact test (Monte Carlo correction)	0.733
BMI			30.7233 (5.19087)	28.9918 (5.73042)	Spearman (-0.097)	0.225
Age			60.88 (6.402)	60.76 (6.774)	Spearman (0.051)	0.522
Postmenopausal age			16.50 (10.309)	13.76 (5.915)	Spearman (0.062)	0.516
Pack-year			4.65625 (5.926541)	8.29286 (9.334510)	Spearman (0.015)	0.923
Number of deliveries		2.82 (1.471)	3.84 (1.928)	3.75 (2.301)	Spearman (0.063)	0.474
Number of kids		2.82 (1.471)	3.84 (1.928)	3.75 (2.301)	Spearman (0.063)	0.474

Correlations between clinical factors and T-scores

Correlation analyses were performed to investigate relationships between selected clinical variables and both spinal and femoral T-scores. Age was not significantly correlated with spinal T-score (Spearman's ρ = 0.034, p = 0.672) or femoral T-score (ρ = 0.022, p = 0.779), indicating negligible effect sizes. Likewise, postmenopausal duration demonstrated no significant correlation with spinal T-score (ρ = -0.016, p = 0.867) or femoral T-score (ρ = 0.143, p = 0.133).

Reproductive history variables, including number of pregnancies and number of children, were not significantly associated with either spinal T-score (ρ = 0.065, p = 0.459 for both variables) or femoral T-score (ρ = 0.083, p = 0.345 for both variables). Smoking exposure measured by pack-years also showed no meaningful correlation with spinal T-score (ρ = 0.006, p = 0.965) or femoral T-score (ρ = 0.089, p = 0.553).

BMI demonstrated the strongest observed relationship with BMD measurements. Although the correlation between BMI and spinal T-score was not statistically significant (Spearman's ρ = -0.131, p = 0.103), BMI was significantly negatively correlated with femoral T-score (Spearman's ρ = -0.257, p = 0.001). According to conventional interpretation criteria, this represents a small-to-moderate effect size (ρ² = 0.066), indicating that higher BMI values were associated with lower femoral T-scores in this cohort.

**Figure 3:**
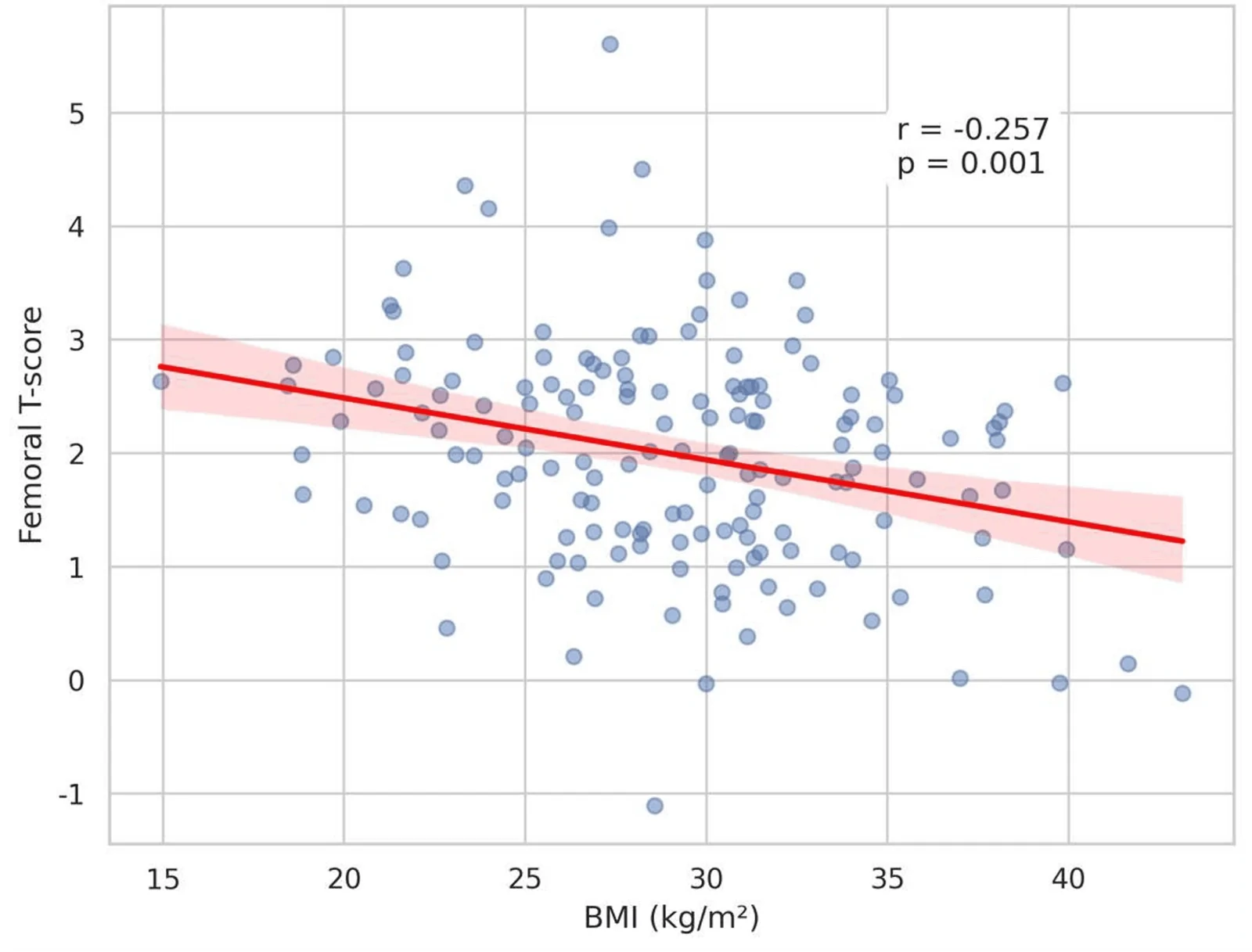
Scatter plot with the regression line showing a significant negative correlation between the body mass index (BMI) and femoral T-score.

Overall, the most examined demographic and clinical variables demonstrated negligible correlations with BMD measurements. The only statistically significant correlation identified was the inverse relationship between BMI and femoral T-score (r = -0.257, p = 0.001) (Figure 3, Table 3).

**Table 3:** Factors correlations with T-scores BMI: body mass index; T-score: standard deviation from the mean bone mineral density of a healthy young adult reference population; Z-score: standard deviation from the age- and sex-matched reference population

	Spine T-score		Femur T-score	
	Factor	P-value	Factor	P-value
Age*	0.034	0.672	0.022	0.779
Postmenopausal years	-0.016	0.867	0.143	0.133
Number of deliveries	0.065	0.459	0.083	0.345
Number of kids	0.065	0.459	0.083	0.345
Pack-year smoking	0.006	0.965	0.089	0.553
BMI*	-0.131	0.103	-0.257	0.001

## Discussion

This cross-sectional study with prospective data collection at the National Hospital in Damascus, Syria, provides the first comprehensive evaluation of osteoporosis prevalence and determinants in a Syrian population undergoing DXA scanning. Our findings reveal a substantial burden of low bone mass, with osteoporosis affecting 53.2% of participants and osteopenia present in 36.7%, while only 10.1% maintained normal bone density. These rates are considerably higher than those reported in many international studies and underscore the urgent need for enhanced osteoporosis screening and management strategies in this setting.

The prevalence of osteoporosis observed in our cohort (53.2%) exceeds the global average of approximately 18-38% reported in systematic reviews [6]. This elevation may be attributed to several factors, including the demographic characteristics of our predominantly female, postmenopausal population, and potential delays in diagnosis and treatment in the context of healthcare system strain during the Syrian conflict. In neighboring Saudi Arabia, studies have reported osteoporosis prevalence ranging from 23.4% to 39.5% in general populations [8,9], with Al-Ahsa showing 31.54% overall [7] and Alkhobar demonstrating 63.6% in men and 52.8% in women when assessed using lumbar spine T-scores [8]. Similarly, research from Pakistan utilizing DXA found an overall prevalence of 38.5%, with site-specific rates of 38.7% in the lumbar spine and 38.4% in the forearm [11]. Our finding of higher osteoporosis prevalence in the Syrian population compared to these regional estimates may reflect differences in study populations, with our cohort comprising patients specifically referred for DXA evaluation, who likely had higher clinical suspicion for bone disease compared to community-based samples.

The striking sex disparity observed in our study, with females comprising 95.2% of the osteoporosis group compared to 75.0% of the normal group (p = 0.003), aligns with established epidemiological patterns [6,7,12]. This female predominance is attributed to the accelerated bone loss during and following menopause, with women experiencing approximately a 10% reduction in BMD during the menopausal transition, and nearly half demonstrating up to 20% loss over five to seven years [15]. The higher prevalence among women in our cohort (86.7% of the total participants) reflects both the biological vulnerability of females to osteoporosis and potential referral patterns, as clinicians may be more likely to request DXA screening for women presenting with risk factors. International data consistently demonstrate higher osteoporosis prevalence in women (35.3%) compared to men (12.5%) [6], a pattern confirmed by recent Saudi Arabian studies reporting 36.7% in women versus 21.2% in men [7].

Although postmenopausal women represented 81% of our study population and were more frequently represented in the osteopenia (82.2%) and osteoporosis (83.8%) groups than in the normal group (58.3%), this difference did not reach statistical significance (p = 0.108). This finding may reflect the relatively small sample size of the normal group or the influence of other factors on BMD status. The mean postmenopausal duration of 14.52 ± 7.29 years indicates prolonged estrogen deficiency exposure, a well-established risk factor for osteoporosis [15]. Interestingly, age at menopause and number of deliveries did not demonstrate significant associations with osteoporosis classification in our bivariate analyses, suggesting that other factors may exert greater influence on bone health in this population.

The association observed between marital status and osteoporosis classification should be interpreted cautiously because the number of unmarried participants was relatively small. Although single individuals represented a higher proportion of the osteoporosis group compared with the osteopenia group, the normal BMD group also showed the highest proportion of single participants. Therefore, marital status may reflect underlying social, lifestyle, or healthcare-related factors rather than a direct biological determinant of bone health. The lack of significant association between the BMI and osteoporosis classification (Spearman's p = -0.097, p = 0.225) contrasts with previous studies demonstrating inverse relationships between BMI and osteoporosis risk [7,11]. In Al-Ahsa, Saudi Arabia, patients with osteoporosis (T-score ≤ -2.5) exhibited the lowest mean BMI (29.1 ± 5.9), while those with normal BMD had the highest (31.8 ± 5.9) [7]. Similarly, a large Irish cohort study found that higher BMI was associated with greater BMD at the total hip in both men and women [3]. The weaker relationship observed in our study may be attributed to the relatively limited BMI range in our population or the predominance of overweight/obese individuals (mean BMI 29.47 ± 5.55 kg/m²), potentially masking the protective effect of higher body weight against bone loss. Alternatively, the high prevalence of other risk factors in our cohort may have overwhelmed the BMI-BMD relationship.

Among the assessed comorbidities, hyperthyroidism demonstrated a significant association with osteoporosis classification (p = 0.025), with the highest prevalence observed in the osteoporosis group (23.8%) compared with the normal (12.5%) and osteopenia (6.9%) groups. This finding is consistent with the well-established relationship between thyroid hormone excess and accelerated bone turnover, leading to reduced BMD and increased fracture risk [16]. Hypothyroidism showed a borderline association (p = 0.053), with prevalence increasing from 0% in the normal group to 20.5% in the osteoporosis group. The relationship between hypothyroidism and bone health is more complex, as thyroid hormone replacement therapy, particularly when over-replaced, may have deleterious effects on bone [16]. The observed trend toward increased osteoporosis prevalence among patients with hypothyroidism in our study warrants further investigation, particularly regarding medication dosing and duration. Surprisingly, we found no significant associations between osteoporosis and other comorbidities including hypertension (p = 0.958), diabetes mellitus (p = 0.452), heart disease (p = 0.128), asthma (p = 0.281), or steroid use (p = 0.679). These findings may reflect the relatively small sample size for subgroup analyses or the possibility that other risk factors in our population supersede the effects of these comorbidities.

Cervical and lumbar disc disease demonstrated unexpected associations with bone density classification. Cervical disc disease was significantly more prevalent among participants with osteopenia (12.1%) compared to those with osteoporosis (2.4%) (p = 0.029), and lumbar disc disease showed highest prevalence in the osteopenia group (22.4%) compared to normal (6.3%) and osteoporosis (6.0%) groups (p = 0.009). These paradoxical associations may reflect diagnostic biases, as patients with disc disease and back pain may be more likely to undergo DXA screening, potentially identifying osteopenia before progression to osteoporosis. Alternatively, these findings could result from the complex interplay between degenerative joint disease and bone metabolism, as osteoarthritis has been associated with higher BMD in some studies [17].

Correlation analyses revealed an unexpected significant inverse relationship between BMI and femoral T-score (r = -0.257, p = 0.001), indicating that higher BMI was associated with lower femoral BMD. This finding contrasts with the generally accepted protective effect of higher body weight on bone health [7] and may reflect the unique characteristics of our population. The discordance between the lack of association between BMI and osteoporosis classification and the significant correlation with femoral T-score may indicate site-specific effects of BMI on bone density, with the femoral neck being more sensitive to mechanical loading and metabolic influences than the lumbar spine [17]. Alternatively, this finding could be influenced by confounding factors such as age, menopausal status, or physical activity levels not captured in our analysis. The weak, non-significant correlations between age, postmenopausal duration, smoking exposure, reproductive history, and T-scores suggest that these factors alone are insufficient to explain the high osteoporosis prevalence in our cohort, emphasizing the multifactorial nature of bone health.

The inverse relationship between BMI and femoral BMD deviates from the established paradigm that higher body weight confers skeletal protection through increased mechanical loading and higher circulating estrogen levels [8]. Several factors may explain this unexpected finding.

First, BMI composition may differ substantially in our population compared to Western cohorts where the protective effect has been primarily documented. BMI does not distinguish between fat mass and lean mass, and visceral adipose tissue may exert negative effects on bone through pro-inflammatory mechanisms [17]. The mean BMI of 29.47 ± 5.55 kg/m² in our cohort indicates a predominantly overweight population, potentially reflecting a higher proportion of adiposity, although body composition and fat distribution were not assessed in this study.

Second, the duration of obesity may be critical. While obesity in younger adults is associated with higher peak bone mass, obesity in later life may not confer the same protective benefits [15]. Given the older age distribution of our cohort (mean age 60.79 years), the protective effects of obesity observed earlier in life may not necessarily apply to this population.

Third, the site-specific nature of our finding suggests that the femoral neck, which has a different cortical-to-trabecular bone composition compared with the lumbar spine, responds differently to mechanical loading and metabolic influences [18].

The high prevalence of osteopenia (36.7%) in our population is particularly concerning, as individuals with osteopenia are also at increased risk for fragility fractures [18]. Early identification of osteopenia provides an opportunity for preventive interventions to delay progression to osteoporosis. Our finding that only 43.0% of participants received vitamin D supplementation and 15.2% received calcium supplementation suggests substantial gaps in preventive care, consistent with observations in other Middle Eastern populations [7]. In Al-Ahsa, treatment rates were notably low at 11.49% among diagnosed osteoporosis patients [7], highlighting a global challenge in osteoporosis management.

Strengths and limitations

This study possesses several strengths. To our knowledge, limited data are available regarding osteoporosis prevalence assessed by DXA in Syrian populations, and this study addresses a critical gap in regional epidemiological data. The prospective design allowed for systematic data collection and complete capture of relevant variables. The use of standardized WHO criteria for osteoporosis classification and appropriate statistical approaches strengthens the interpretation of our findings. The inclusion of both lumbar spine and femoral neck measurements, with calculation of the lowest T-score for classification, aligns with International Society for Clinical Densitometry (ISCD) recommendations [19].

Several limitations warrant consideration. First, a formal sample size calculation was not performed because all eligible patients during the study period were consecutively included. However, the relatively limited sample size may have reduced the statistical power to detect some associations, increasing the possibility of type II error. Second, the cross-sectional design precludes causal inferences regarding the relationships between risk factors and osteoporosis. Third, the study population comprised patients referred for DXA scanning, introducing selection bias and limiting generalizability to the general Syrian population. Patients referred for DXA are more likely to have risk factors for osteoporosis, potentially overestimating prevalence compared to community-based samples. Fourth, the predominantly female population (86.7%) limits our ability to draw robust conclusions regarding osteoporosis in Syrian men. Fifth, missing data for some variables, including lifestyle factors and medication details, may have reduced statistical power for certain analyses. Sixth, we did not assess additional important determinants of bone health, such as dietary calcium intake, physical activity levels, sun exposure, or biochemical markers of bone turnover. Seventh, the use of a single DXA machine at one center may limit the generalizability of our findings to other settings in Syria. Eighth, BMD classification was based on measurements from the lumbar spine and femoral neck only, as the DXA scanner available at our center did not provide total hip measurements during the study period; this may limit comparability with recommendations that incorporate additional skeletal sites. Ninth, multiple bivariate analyses were conducted without adjustment for multiple comparisons; therefore, the significant associations identified should be interpreted cautiously. Finally, we did not evaluate fracture history or future fracture risk using validated tools such as FRAX, which would provide additional clinical context for the BMD findings.

Clinical and public health implications

The findings of this study have important implications for clinical practice and health policy in Syria. The high prevalence of osteoporosis and osteopenia among patients undergoing DXA scanning underscores the urgent need for enhanced screening programs targeting high-risk populations, particularly postmenopausal women and patients with thyroid disorders. Given the limited DXA availability in Syria, resource-appropriate screening strategies, such as the use of validated risk assessment tools, should be considered to identify individuals who would most benefit from DXA referral. The identification of hyperthyroidism as a significant risk factor suggests that patients with thyroid disorders should be prioritized for bone health assessment. The low rates of calcium and vitamin D supplementation indicate opportunities for improved preventive care, including provider education and public health campaigns emphasizing bone-healthy nutrition and lifestyle.

The substantial treatment gap observed in our study, with only 43.0% receiving vitamin D and 15.2% receiving calcium supplementation, reflects a broader challenge in osteoporosis management in the region [7]. Strengthening primary care management of osteoporosis, including implementation of fracture liaison services, could improve identification and treatment of patients at risk [18]. Policy changes to expand access to DXA scanning and osteoporosis medications are essential, particularly in the context of the Syrian healthcare system's recovery and rebuilding efforts.

Future research directions

Future research should address the limitations of this study through population-based investigations to establish the true prevalence of osteoporosis in the Syrian general population. Longitudinal studies are needed to evaluate the progression from osteopenia to osteoporosis and to identify modifiable risk factors amenable to intervention. Research incorporating fracture outcomes would help establish the clinical significance of DXA findings in the Syrian context. Evaluation of the performance of fracture risk assessment tools, such as FRAX, in the Syrian population would facilitate evidence-based clinical decision-making. Additionally, studies examining the acceptability, feasibility, and cost-effectiveness of various screening strategies in resource-limited settings would inform health policy development. Finally, investigation of the impact of conflict-related factors, including displacement, food insecurity, and healthcare disruption, on bone health would provide insights into the unique challenges facing this population.

## Conclusions

This cross-sectional study with prospective data collection reveals a substantial burden of osteoporosis and osteopenia among patients undergoing DXA scanning at the National Hospital in Damascus, Syria, with 53.2% meeting the WHO criteria for osteoporosis. Female sex and hyperthyroidism were identified as significant risk factors. The high prevalence of low bone mass and low rates of preventive interventions underscore the urgent need for enhanced screening programs and public health strategies targeting osteoporosis in Syria. These findings contribute to the limited epidemiological data on osteoporosis in conflict-affected settings and have important implications for clinical practice and health policy in the region. Future research should focus on population-based investigations and evaluation of fracture outcomes to guide evidence-based osteoporosis management in Syria.
